# Evaluation des pratiques de l’automédication et leurs caractéristiques auprès des étudiants d’Uvira en République Démocratique du Congo

**DOI:** 10.11604/pamj.2023.45.53.39690

**Published:** 2023-05-23

**Authors:** Henry Manya Mboni, Arsène Kabamba Tshikongo, Valentin Bashige Chirubagula, Cedrick Mutombo Shakalenga, Arsène Mutula Kanyegere, Bontemps Byakujoga Rugema, Saili Stay Mushobekwa, Derrick Bushobole Akiba, Nicolas Mihuhi Rusati

**Affiliations:** 1 Département de Pharmacologie, Faculté des Sciences Pharmaceutiques, Université de Lubumbashi, Lubumbashi, République Démocratique du Congo,; 2 Filière des Techniques Pharmaceutiques, Institut Supérieur des Techniques Médicales d´Uvira, Uvira, République Démocratique du Congo,; 3 Département de Biologie Clinique, Faculté des Sciences Pharmaceutiques, Université de Lubumbashi, Lubumbashi, République Démocratique Congo,; 4 Filière des Sciences Infirmières, Institut Supérieur des Techniques Médicales d´Uvira, Uvira, République Démocratique du Congo,; 5 Filière de Santé Publique, Institut Supérieur des Techniques Médicales d´Uvira, Uvira, République Démocratique du Congo

**Keywords:** Automédication, milieux universitaires, étudiants, Uvira, Self-medication, academia, students, Uvira

## Abstract

**Introduction:**

actuellement, l´automédication constitue une menace de la santé publique. Cette étude avait pour objectif d´évaluer les pratiques de l´automédication chez les étudiants d´Uvira en République Démocratique Congo.

**Méthodes:**

il s´agit d´une étude descriptive transversale menée sous forme d´interview indirecte auprès de 700 étudiants grâce à un questionnaire d´enquête auto-administré. Les données ont été traitées avec XLSTAT.

**Résultats:**

sur les 700 étudiants enquêtés, la prévalence de l´automédication représente 99,3% (n=695) dont 42,3% (n=294) l´ont débutée à l´adolescence. Du total, 57,4% (n=399) certifient faire recours à l´automédication chaque fois qu´ils tombent malades et qu´ils sont en manque d´argent (n=471, 67,7%) pour une consultation; la pathologie la plus citée au cours de cette pratique étant le paludisme (n=212, 30,5%). De tous les médicaments recourus, le Paracétamol occupe la première place (n=106, 15,3%) alors que le comprimé constitue la forme la plus usée par ces étudiants (n=598, 86%). Sur le plan de l´association médicamenteuse, on note en nombre de fréquence élevée le Fansidar-Coartem (n=106, 17,2%); la posologie dépendant de l´âge dans 65,6% (n=456) de cas. L´étude a aussi démontré que 37,4% (n=695) des étudiants interviewés recourent à la phytothérapie, surtout pour traiter le paludisme (n=124, 47,3%).

**Conclusion:**

chez les étudiants d´Uvira, l´automédication est extrêmement pratiquée, surtout contre le paludisme. Des efforts dans la sensibilisation à l'usage rationnel des médicaments sont à entreprendre par les personnels de santé en connivence avec les décideurs politiques pour mieux contrôler voire éradiquer cette pratique néfaste à la santé.

## Abstract (en)

**Introduction:**

self-medication poses a threat to public health. The purpose of this study was to evaluate self-medication practices among Uvira students in the Democratic Republic of the Congo (DRC).

**Methods:**

we conducted a cross-sectional descriptive study based on an indirect interview of 700 students, using a self-administered survey questionnaire. Data were processed using XLSTAT.

**Results:**

according to the survey conducted on these 700 students, the prevalence of self-medication was 99.3% (n=695), of which 42.3% (n=294) started during adolescence. Of the total respondents, 57.4% (n=399) said they resorted to self-medication every time they fell ill and were short of money (n=471, 67.7%) for a consultation. The most commonly cited pathology encountered during this practice was malaria (n=212, 30.5%). Of all drugs used, paracetamol was in the first place (n=106, 15.3%) while the tablet was the form most used by these students (n=598, 86%). In terms of the drug combination, Fansidar-Coartem (n=106, 17.2%) was the most common; the dosage was age-dependent in 65.6% (n=456) of cases. This study also showed that 37.4% (n=695) of the students interviewed used phytotherapy, mainly to treat malaria (n=124, 47.3%).

**Conclusion:**

among Uvira students, self-medication is extremely used, especially against malaria. Efforts must be made by healthcare personnel in collaboration with policymakers to raise awareness about the rational use of medications, in order to better control and potentially eradicate this practice that is harmful to health.

## Introduction

L´administration de tout médicament (que ça soit d´origine de la médecine moderne ou celle traditionnelle) de son propre gré ou de celui d´un familier afin de soigner une affection ou un symptôme identifié par lui-même, sans consulter un professionnel de santé, s´appelle l'automédication [1-3]. En effet, les raisons courantes de l´automédication incluent les plus souvent le coût élevé de prise en charge des malades dans les formations sanitaires, le faible pouvoir d´achat, l´insuffisance en infrastructures et personnels de santé, la banalisation de certaines maladies, le mépris des règles de délivrances des médicaments par certains vendeurs en pharmacie et les faibles connaissances de la population sur les risques encourus aux mauvais usages des médicaments et à la non-maitrise des indications, des contre-indications, des posologies, des rythmes d´administration et la durée du traitement [4-6]. Ainsi, cette pratique est dangereuse pour la santé publique et peut entraîner les plus souvent des résistances microbiennes acquises envers les médicaments, surtout les antibiotiques, les accidents médicamenteux par surdosage et/ou des allergies, les interactions médicamenteuses non bénéfiques, la pharmacodépendance et la toxicomanie [7,8]. En République Démocratique du Congo, la prévalence de l´automédication a été estimée à 49% en 2005, sur l´ensemble de la population congolaise [6]. Elle a été de 5,6% chez des patients reçus aux urgences médicales des Cliniques Universitaires de Kinshasa en 2011 [9], de 57% au sein de la population de Goma en 2013 [10], de 99% chez les étudiants de l´Université de Lubumbashi en 2015 [6,11], et de 61,3% chez les femmes enceinte de Bukavu en 2016 [12]. Néanmoins dans la ville d´Uvira, des données relatives à la prévalence et aux caractéristiques de l´automédication au sein de la population en général et plus particulièrement chez les étudiants restent inconnues; d´où, la présente étude a été initiée en vue de déterminer le taux de cette pratique et les facteurs y associés auprès des étudiants fréquentant les différents instituts supérieurs et universités de la ville d´Uvira dans la province de Sud-Kivu en République Démocratique du Congo.

## Méthodes

**Type, lieu et conception de l'étude:** il s´agit d´une étude descriptive transversale analytique effectuée entre les mois de septembre 2020 et août 2021 afin de déterminer par une technique d'échantillonnage aléatoire la fréquence de l´automédication chez les étudiants des institutions de l´enseignement supérieurs et universitaires ci-après: (i) FBN Universty of Uvira, (ii) Institut Supérieur Technologique d´Uvira (IST), (iii) Institut Supérieur de Commerce d´Uvira (ISC), (iv) Institut Supérieur des Techniques Médicales d´Uvira (ISTM), (v) Institut Supérieur de Développement Rural d´Uvira (ISDR), (vi) Institut Supérieur Pédagogique d´Uvira (ISP), (vii) Université Libre d´Uvira et de Grands Lacs (ULUGL), (viii) Université de Grands Lacs Africains d´Uvira (UGLA), (ix) Université Francophone d´Uvira (UFU) et (x) Université Notre Dame de Tanganyika (UNDT). Ces institutions se situent dans la ville d´Uvira qui se localise dans la province du Sud-Kivu en République Démocratique du Congo aux coordonnées GPS: S 03°26', E 29°08' [13]. Menée sous forme d´interview indirecte, l´enquête a eu recours à un questionnaire préétabli reprenant essentiellement les questions en rapport avec l´usage des médicaments sans prescriptions, les raisons de cette pratique, la dernière période de l´automédication, les maladies, les risques, les médicaments utilisés, la posologie, le recours à la phytothérapie et les données concernant les informateurs. Ce questionnaire a été établi après une revue systématique dans différents moteurs de recherche (Google Scholar, HINARI, Medline/ PubMed et Science Direct) sur les travaux antérieurs en rapport avec la prévalence de l´automédication dans les milieux universitaires.

**Population de l´étude:** l´étude a concerné les étudiants inscrits régulièrement au courant de l´année académique 2020-2021 dans dix institutions publiques et privées d´enseignements supérieurs et universitaires de la ville d´Uvira. Selon les différents registres des services de secrétariats généraux de ces institutions, un total de 2277 étudiants étaient inscrits régulièrement au courant de l´année académique de cette étude. Pour chacune de ces institutions, les étudiants ont été sélectionnés aléatoirement. Seuls les étudiants disponibles au moment de l´enquête ayant donné leur consentement à participer dans l´étude ont été inclus. La non-inclusion a concerné tout étudiant involontaire et non disponible dans ces institutions au moment de l´enquête.

**Taille d´échantillon:** elle a été déterminée à l´aide d´un calculateur implémenté dans un site web. Considérant la taille de la population à l´étude (2277 étudiants), la marge d´erreur (5%) et le niveau de confiance (95%), la taille minimale d´échantillon serait de 329 étudiants. Les étudiants ont été ciblés aléatoirement pour participer à notre enquête; avec les explications sur les objectifs de l´étude, l´utilisation des données et la liberté de participer ou non à l´étude, seuls ceux (773 étudiants) ayant accepté d´être interviewés ont fait partie de notre panel.

**Collecte des données:** les données ont été collectées à l´aide d´un questionnaire auto administré, composé de 22 questions. Le questionnaire a été adapté et validé après une pré-enquête auprès de 25 sujets dans la population cible. Parmi les 773 questionnaires complétés, 73 ont été écartés car mal complétés, ce qui a réduit la taille de l´échantillon à 700 étudiants. Les données issues de ces étudiants ont été par la suite enregistrées dans une feuille de calcul Excel.

**Analyse des données:** les données collectées ont été soumises au logiciel XLSTAT (Version 2023.1.2./1406). Le calcul des fréquences a été effectué pour les variables qualitatives alors que les calculs de la moyenne et de l´écart-type ont concerné les variables quantitatives. La prévalence de l´automédication a été également calculée et exprimée en pourcentage. Pour la comparaison entre les paramètres socio-démographiques et la prévalence de l´automédication, le test de chi carré a été établi et le p-value <0,05 a constitué le seuil de décision.

**Considérations éthiques:** les participants avaient été préalablement informés sur le but de l´étude et, sur la confidentialité et l´anonymat des informations à recueillir. Le consentement libre et éclairé de chaque participant était scrupuleusement respecté. Signalons également que l´étude avait reçu les autorisations verbales des responsables des institutions concernées et avait été menée conformément aux directives éthiques de la Déclaration d'Helsinki.

## Résultats

La présente étude a été menée auprès de 700 étudiants d´âge oscillant entre 18 et 46 ans dont la moyenne d´âge était de 24,8±6,3 ans. La majorité des enquêtés avaient l´âge compris entre 18 et 20 ans (44,9%), avec un sexe ratio de 1,6 en faveur des femmes (F/H). De tous les enquêtés, il apparait que 296 sujets (42,3%) étaient dans les sciences de la santé contre 404 (57,7%) qui n´en étaient pas. La prévalence de l´automédication dans cette population estudiantine représente un taux de 99,3%, ce qui veut simplement dire que tous avaient déjà recouru au moins une fois à l´automédication; seul 0,7% consulte un personnel de santé avant toute prise de médicament (Tableau 1). La grande majorité (57,4%) faisait en premier lieu recours à cette pratique chaque fois qu´elle tombait malade. Vingt-huit virgule cinq pourcent (28,5%) y font recours dans certains cas spécifiques alors que 14,1% ne les faisaient que très rarement. Dans ces scénarios, le manque d´argent pour consulter (67,7%) constitue la principale raison les poussant à s´automédiquer. Et c´est pour lutter contre plusieurs pathologies/ou symptômes dont les plus fréquents sont constitués du paludisme (30,5%), des céphalées (18,1%), de la fièvre typhoïde (9,4%), des dysménorrhées (6,8%) et des douleurs abdominales (6,6%) (Tableau 2). Cependant, les enquêtés avaient confirmé à 97% avoir conscience des risques encourus en pratiquant l´automédication; parmi eux, 40,4% estimaient que ce risque possible pourrait être lié au traitement inadapté, 30,6% à une erreur de dose, 13,6% à une erreur de diagnostic et 9,9% à des interactions médicamenteuses (Tableau 2).

**Tableau 1:** paramètres sociodémographiques et pratique de l’automédication

Variables	Automédication (N=695)		Pas d’automédication (N=5)		Total (N=700)	p-value
**Genre**						
Homme	268(98,9%)		3(1,1%)		271(38,7%)	0,326
Femme	427(99,5)		2(0,5%)		429(61,3%)	
**Tranche d’âge**						
[18-20]	307(99%)		3 (1%)		310 (44,3%)	0,728
[20-30]	249(99,2%)		2(0,8%)		251(35,9%)	
[30-40]	134(100%)		0(0%)		134(19,1%)	
[40-46]	5(100%)		0(0%)		5(0,7%)	
**Domaine d’étude**						
**Santé**						
1^er^ cycle	271(98,5%)		4(1,5%)		275(39,3%)	0,578
2^e^ cycle	21(100 %)		0(0%)		21(3%)	
**Autres**						
1^er^ cycle	278(99,6%)		1(0,4%)		279(39,8%)	0,503
2^e^ cycle	125(100 %)		0(0%)		125(17,9%)	
						

**Tableau 2:** motifs, pathologies ou symptômes conduisant à l’automédication et risques encourus (N=695)

Variables	Ni (%)
**Dans quelles circonstances vous recourez à l’automédication ?**	
Chaque fois que vous tombez malade	399 (57,4)
Dans certains cas	198 (28,5)
Très rarement	98 (14,1)
**Pour quelles raisons vous faites recours à l’automédication ?**	
Brusquement arrivé	20(2,9)
Habitude	37(5,3)
La garantie de Dieu	11 (1,6)
Manque d'argent pour voir le médecin	471 (67,7)
Mauvais état de santé	17 (2,4)
En cas de maladie moins grave	64 (9,2)
Temps long d'attente lors de la consultation	49 (7)
Connaissance en médecine étant étudiant en médecine	21(3)
Sur base d´un bon médical antérieur	1(0,1)
Jamais tombé malade	3 (0,4)
L'automédication coute moins cher	1 (0,1)
**Pathologies ou symptômes**	
Paludisme	212 (30,5)
Céphalée	126 (18,1)
Fièvre typhoïde	65 (9,4)
Infection	35 (5)
Dysménorrhée	47 (6,8)
Douleurs abdominale	46 (6,6)
Asthénie	23 (3,3)
Gastrite	14(2)
Grippe	21(3)
Angine	11(1,6)
Toux	9(1,3)
Diarrhée	6(0,9)
Fièvre	37(5,3)
Acné	1(0,1)
Plaie	1(0,1)
Fatigue	6(0,9)
Rhume	3(0,4)
Maux de ventre	30(4,3)
Autres	2(0,3)
**Connaissance des risques encourus lors de l´automédication**	
Oui	674(97)
Non	21(3)
**Types de risque de l´automédication (N=674)**	
Traitement inadapté	272(40,4)
Erreur du diagnostic	92(13,6)
Erreur de la dose	206(30,6)
Interaction médicamenteuse	67(9,9)
Allergie	8(1,2)
Aggravation des signes ressentis	9(1,4)
Autres	20(3)

Par rapport à l'âge de la première automédication, les sujets avaient été groupés en 5 classes (de 10 à 14 ans, 15 à 19 ans, 20 à 24 ans, 25 à 29 ans et 30 à 46 ans) en fonction des étapes du développement psychosocial de la personnalité selon Erickson [14]. Il en découle que 42,3% des sujets avaient commencé leur automédication entre 10 et 14 ans, 37,4% entre 15 et 19 ans, 9,2% entre 20 et 24 ans, 5,5% entre 30 et 46 ans, et 5,2% entre 25 et 29 ans. En plus, à plus ou moins un mois de notre enquête, 67,3% des étudiants enquêtés avaient eu recours à l´automédication. Parmi les sujets qui s´automédiquent, 68,9% s´étaient initiés seuls à cette pratique, et leurs principales sources d´information au cours de cette pratique étaient des vendeurs en pharmacie (28,8%), les conseils de leurs collègues étudiants du domaine de la santé (25,8%), la lecture des notices des médicaments (21,2%) et leur entourage (21%) (Tableau 3). Voulant traiter leurs maladies, les enquêtés recourent aux divers médicaments en automédication. De ceux-ci, les plus fréquents étaient le Paracétamol (15,3%) et le Coartem (13,2%). Notons également que 88,5% des étudiants associaient plusieurs médicaments lors de leur pratique d´automédication. Dans leur association médicamenteuse, on note successivement Fansidar-Coartem (17,2%), Paracétamol-Action (11,2%), Coartem-Quinine (9,6%), Tinidazole-Ciprofloxacine (4,4%), Bactrim-Chlorphéniramine (3,4%) et Amoxicilline-ciprofloxacine (3,1%). De tous ceux qui avaient appliqué au moins une fois une association médicamenteuse, 70,4% des sujets affirment s´être ressourcés auprès des pharmaciens. S´agissant des formes des médicaments les plus utilisés par les étudiants, les comprimés en étaient usés majoritairement (86%) (Tableau 4).

**Tableau 3:** âge de la première automédication, période du dernier recours, instigateur et source d´informations en rapport avec cette pratique (N= 695)

Variables	Ni (%)
**Âge (ans)**	
10 à 14	294 (42,3)
15 à 19	260(37,4)
20 à 24	64(9,2)
25 à 29	36(5,2)
30 à 46	38(5,5)
Rien à signaler	3(0,4)
**Période**	
≤ 1 mois	468(67,3)
> à 1 mois	225 (32,4)
Je ne me rappelle pas	2(0,3)
**Instigateur de l´automédication**	
Vous-même	479(68,9)
Un proche	125(18)
Un parent	71(10,2)
Un connaisseur	15(2,2)
Rien à signaler	5(0,7)
**Sources des informations en rapport avec la pratique de l´automédication**	
Entourage	146(21)
Etudiants en médecine ou sciences de la santé	179(25,8)
Notice des médicaments	147(21,2)
Vendeurs dans une pharmacie	200(28,8)
Internet	12(1,7)
Rien à signaler	11(1,6)

**Tableau 4:** médicaments utilisés au cours de l’automédication

Produits utilisés	Ni (%)	Produits utilisés	Ni (%)	Produits utilisés	Ni (%)
**En cas de monothérapie (N=695)**					
Coartem	92(13,2)	Cloxacilline	10(1,4)	Nystatine	3(0,4)
Ciprofloxacine	79(11,4)	Diclofénac	22(3,2)	Oméprazole	3(0,4)
Paracétamol	106(15,3)	Bactrim	8(1,2)	Phosphalugel	3(0,4)
Quinine	40(5,8)	Céfixime	7(1)	Doliprane	2(0,3)
L-Artem	48(6,9)	Doxycycline	7(1)	Lasix	2(0,3)
Métronidazole	46(6,6)	Chlorphéniramine	3(0,4)	Motilium	2(0,3)
Ibuprofène	35(5)	Tétracycline	10(1,4)	Epiderme	1(0,1)
Art quick	26(3,7)	Dolaren	6(0,9)	Pénicilline v	1(0,1)
Amoxicilline	15(2,2)	Dr cold	5(0,7)	Pilorymex	1(0,1)
Fansidar	14(2)	Gogynax	5(0,7)	Hedone	6 (0,9)
Action	24(3,5)	Artésunate	3(0,4)	Loprade	6(0,9)
Dr Maison	13 (1,9)	Chloramphénicol	3 (0,4)	Autres	10 (1,4)
C-Trl	11 (1,6)	Dexaméthasone	3 (0,4)		
Papavérine	11 (1,6)	Gélusile	3 (0,4)		
**Formes médicamenteuses utilisées (N=695)**					
**Variables**		**Ni (%)**	**Variables**		**Ni (%)**
Comprimé		598 (86)	Suspension		3 (0,4)
Capsule		23 (3,3)	Soluté Massif		3 (0,4)
Ovule		28 (4)	Suppositoire		2 (0,2)
Gélule		26 (3,7)	Pilule		1 (0,1)
Sirop		9 (1,2)	RAS		2 (0,2)
**En cas de polythérapie (N=695)**					
**Avez-vous déjà fait recours à une association des médicaments dans le cadre de l’automédication ?**					
Oui			615 (88,5)		
Non			80 (11,5)		
**Quelles sont les combinaisons médicamenteuses utilisez-vous? (N=615)**					
**Médicaments associés**	**Ni (%)**	**Médicaments associés**	**Ni (%)**	**Médicaments associés**	**Ni (%)**
Fansidar-Coartem	106(17,2)	Paracétamol-Dr cold	5(0,8)	Métronidazole-Loprade	2(0,3)
Paracétamol-Action	69 (11,2)	Zendex-Aspirine	4 (0,7)	Papavérine-Métronidazole	2 (0,3)
Coartem-Quinine	59 (9,6)	Action-Méprobamate	3 (0,5)	Paracétamol-Diclofénac	2 (0,3)
Tinidazole-Ciprofloxacine	27 (4,4)	Ampicilline-Vitamine B complexe	3 (0,5)	Action-Aspirine	1 (0,2)
Bactrim-Chlorphéniramine	21(3,4)	Ciprofloxacine-Coartem	3(0,5)	Amoxicilline-Vitamine C	1(0,2)
Amoxicilline-Ciprofloxacine	19(3,1)	Dexaméthasone-Prométhazines	3(0,5)	Ampicilline-Dexaméthasone	1(0,2)
Ibucap-Muscle plus	13 (2,1)	Dolaren-Métronidazole	3 (0,5)	Chlorphéniramine-paracétamol	1 (0,2)
Paracétamol-Dolaren	13 (2,1)	Amodiaquine-Métronidazole-Paracétamol	3 (0,5)	Coartem- Ciprofloxacine	1 (0,2)
Amoxicilline- Cloxacilline	11(1,8)	Ibuprofène-Paracétamol	17(2,8)	Coartem-Paracétamol	1 (0,2)
Ibuprof&egravte;ne-Diclofénac	10(1,6)	Fansidar-Paracétamol	3(0,5)	Dexaméthasone-Diclofénac	1 (0,2)
Ciprofloxacine-Chloramphénicol	9 (1,5)	Prométhazine-Prednisolone	3 (0,5)	Diclofénac-Hedone	1 (0,2)
Vitamine C- Pénicilline V	9 (1,5)	Tétracycline-Métronidazole	3 (0,5)	Gélusile-Phosphalugel	1(0,2)
Métronidazole-Ciprofloxacine	8(1,3)	Cloxacilline-Vitamine C	2(0,3)	Ibuprofène-paracétamol	1(0,2)
Oméprazole-Phosphalugel	8(1,3)	Dexaméthasone-Cloxacilline	2(0,3)	Paracétamol-Quinine	1 (0,2)
Quinine-Métronidazole	8 (1,3)	Dr maison-Métronidazole	2 (0,3)	Vermox- Métronidazole	1 (0,2)
Bactrim- Cloxacilline	7 (1,1)	Hedone-Diclofénac	2 (0,3)	Métronidazole-Paracétamol- Amoxicilline	3(0,5)
Chloramphénicol- Métronidazole	7(1,1)	Hedone-Doliprane	2(0,3)	Rien à signaler	110(17,9)
Paracétamol-Dexaméthasone	7(1,1)	Indocid-Diclofénac	2(0,3)		
Gélusile-Amoxicilline	6(1)	Métronidazole-Action	2(0,3)		
**Où tirez-vous le savoir pour associer vos médicaments? (N=615)**					
**Variables**		**Ni(%)**	**Variables**		**Ni (%)**
Pharmacien		433(70,4)	Connaisseur en médecine		63(10,2)
Médias		10(1,6)	Autres		10(1,6)
Notices des médicaments		99(16,1)			

Il a été observé que le choix des médicaments à prendre dans le cadre de l´automédication était souvent orienté par une prescription antérieure pour les mêmes symptômes (49,8%) et la posologie était fonction de l´âge (65,6%); la prise des médicaments se faisant le soir (68,2%) presque par tous (Tableau 5). Au tour de 85,5% des étudiants interrogés affirment avoir procédé au changement des médicaments à la suite de leur inefficacité dans 54,9% des cas du premier échec thérapeutique. De tous les enquêtés, 66% certifient qu´ils consultent un médecin en cas d´un double échec thérapeutique, à qui ils signalent (à 94,1%) le fait de s´être automédiqués à domicile (Tableau 6). Les résultats de cette enquête démontrent aussi que 37,4% des étudiants interrogés avaient déjà recouru à la phytothérapie lors de leur pratique de l´automédication et le paludisme à l´échelle de 47,3%, est la pathologie la plus incriminée lors de l´utilisation de ces plantes médicinales en automédication (Tableau 7). Il est important de signifier qu´aucune corrélation ou dépendance statistique (Tableau 1) n´a été mis en évidence notamment par rapport au genre, domaine de formation ou tranche d´âge des personnes interviewées.

**Tableau 5:** facteurs de prédilection et posologie

Variables	Ni (%)	
**Facteurs de prédilection (N=695)**		
Prescription antérieure	346 (49,8)	
Présence de la pharmacie familiale	256 (36,8)	
Conseil du Pharmacien	91(13,1)	
Rien à signaler	2(0,3)	
**Détermination de la posologie (N=695)**		
Age	456(65,6)	
Ancienne ordonnance	136(19,6)	
Poids	37(5,3)	
Importance des signes ressentis	64 (9,2)	
Rien à signaler	2 (0,3)	
**Heure de prise de médicament (N=695)**		
Matin	73(10,5)	
Matin, Midi et soir	96(13,8)	
Matin et soir	11 (1,6)	
Midi	39(5,6)	
Soir	474(68,2)	
Rien à signaler	2 (0,3)	
		

**Tableau 6:** changement des produits, raison de changement et personne consultées en cas d'échec

Variables	Ni (%)
**Avez-vous déjà décidé de changer les produits pendant votre automédication (N=695)**	
Oui	594(85,5)
Non	99 (14,2)
Rien à signaler	2(0,3)
**Raison de changement des produits (N=594)**	
Absence de la guérison	3(0,5)
Accélérer la guérison	6(1)
Lenteur du changement sanitaire	3(0,5)
Médicaments pris n'ont pas bien réagi	3 (0,5)
Pas d´évolution du traitement	54(9,1)
Inefficacité du médicament	326 (54,9)
Effets secondaires	115(19,4)
Rien àsignaler	84(14,1)
**Personnes consultées en cas déinsatisfaction après changement de médicaments (N=594)**	
Un médecin	392(66)
Un Pasteur	85(14,3)
Un pharmacien	113 (19)
Un tradipraticien	4(0,7)
**Indication au médecin des médicaments utilisés après échec thérapeutique (N=594)**	
Oui	559(94,1)
Non	35(5,9)

**Tableau 7:** recours aux plantes médicinales et types des maladies traitées par ces plantes

Variables	Ni (%)
**Avez-vous déjà fait un recours aux plantes médicinales? (N=695)**	
Oui	262(37,4)
Non	433(61,9)
**Pour quel type des maladies? (N=262)**	
Paludisme	124(47,3)
Panaris	6(2,3)
Fracture	5(1,9)
Fièvre	31 (11,8)
Toux	3(1,1)
Accident	2(0,8)
Fièvre typhoïde	23(8,8)
Autres maladies	68(26)

## Discussion

Cette étude, en rapport avec l´automédication chez 700 étudiants de différentes institutions d´Uvira, a révélé une prévalence très élevée (n=695, 99,3%) de cette pratique. Des récentes études effectuées dans différents milieux scolaires et universitaires à travers le monde avaient révélé des prévalences d'automédication variant de 25,2 à 95% [3-5,7,15-23], des taux qui s´avèrent faibles par rapport à ceux de la présente investigation. Ce même constat s´observe par comparaison avec les prévalences de l´automédication trouvées par Eva (49%) à Goma en RDC [24] et ceux de Da Silva (86,4 %) auprès des étudiants des universités de la cité de Rio Grande du Brésil [25]. La disparité de l'organisation du système de santé de ces pays associée aux habitudes socio-culturelles pourrait justifier ces taux variables. En revanche, le pourcentage d´automédication trouvé dans cette étude se révèle proche de celui observé chez les étudiants de l´Université de Lubumbashi en RDC [6] même si les populations d´études de ces deux investigations ne sont pas identiques; nous pensons que cela serait dû au fait que les étudiants se considèrent comme la classe de l´élite de demain, et par conséquent sont capables dès maintenant d´analyser les faits et de prendre la décision sans l´intervention de quelqu´un d´autre. La forte prévalence observée dans ces deux villes de la RDC serait également due à une mauvaise application de la règlementation pharmaceutique. Normalement, les médicaments dangereux et à risque doivent être vendus sur ordonnance, et même la phrase « ne pas vendre sans ordonnance » est parfois étiquetée sur certains des contenants de médicaments [5]. Malheureusement, cette règle n´est pas mise en œuvre par de nombreuses pharmacies pour diverses raisons, en l´occurrence le manque de surveillance adéquate par les autorités concernées et l´aspect financier. Par ailleurs, l´étude menée par [25] démontre que les pratiques d´automédication apparaissent très souvent chez des distributeurs privés et informels des produits pharmaceutiques ; les demandes spontanées de médicaments, sans présentation d´ordonnance ou demande de conseils au vendeur, constituent dans le secteur privé (pharmacies et établissements de vente en gros des produits pharmaceutiques) et informel, la modalité principale d´achats (entre 65 et 73% des ventes observées) [26]. Signalons que dans cette étude, 42,3% des participants étaient inscrits en sciences de la santé. Par comparaison avec la prévalence de la pratique de l´automédication trouvée par cette étude, la situation reste inquiétante au sein de la population enquêtée. Près de la moitié d´entre eux étant du domaine médical, les connaissances acquises sur le risque lié à cette pratique devraient influer en baisse le taux élevé de la prise des médicaments sans ordonnances mise en évidence.

Dans cette étude, nous avons observé une prédominance des femmes (61,3%). Ce constat est presque pareil à d´autres études réalisées en République démocratique du Congo [6,10] et au Nigeria [27] attestant la prédominance du genre féminin, une réalité contradictoire à celle observée au Togo par [28]. En accord avec cette étude, plusieurs recherches révèlent que l´automédication a tendance à augmenter avec l´âge [29-31]. Dans le cadre de cette étude, les étudiants ont décrit comme risques d´automédication un traitement inadapté, une erreur de dose, une erreur de diagnostic et des interactions médicamenteuses. Ces éléments du risque de la pratique d´automédication concordent avec ceux de plusieurs autres études [32,33]. Bien qu´ils soient informés de ces risques, 99,3% y font tout de même régulièrement recours. Le soupçon du paludisme, souvent non confirmé par un diagnostic biologique, constitue la pathologie la plus observée conduisant à 30,5% de nos enquêtés à s´automédiquer; ce résultat corrobore avec une étude réalisée à Lubumbashi, en RD Congo [6] mais est en discordance avec ceux trouvés en Ethiopie [34] et au Rwanda [35] dominés respectivement par les maux de tête (86,5 %) et la fièvre(77,2%). Toutefois, ces deux derniers symptômes sont les plus souvent rencontrés en cas du paludisme [36,37] qui est une parasitose endémique dans la région d'étude [38]. Même si la symptomatologie du paludisme semble être bien connue par la population d´étude [13], le diagnostic biologique avant tout traitement reste important sans lequel la population qui s´automédique effectuerait un traitement inadapté, ce qui pourrait favoriser l'émergence de souches résistantes.

Dans l´ensemble, les étudiants ayant recouru à l'automédication ont avancé plusieurs raisons comme motivation. Les causes fréquentes sont manifestement le manque d´argent pour la consultation (67,7%), les maladies jugées moins grave par eux-mêmes (9,2%) et le long moment d´attente lors de la consultation (7%). Ces allégations sont aussi signalées par plusieurs auteurs comme raisons conduisant à l´automédication [3,11,33,39,40]. Parlant d´un « long moment d´attente », il apparait que l'état socio-économique des sujets possède une implication probable dans cette cause d´automédication [41,42]. Pour les patients ayant un niveau socio-économique élevé, le temps d'attente plus long dans les établissements de santé et le manque de confiance dans les services médicaux sont les facteurs les plus souvent évoqués [43]. Mais cette réalité s´avère non compatible à certains travaux d´autres régions notamment au Burkina-Faso où les raisons apparentes ayant conduit les patients à pratiquer l´automédication se résumaient principalement en une douleur décrite comme insupportable (57,9%) et le manque de moyens financiers pour honorer les frais des traitements (26,3%) [32]. Selon cette étude, le Paracétamol (15,3%) et le Coartem (13,2%) sont les médicaments les plus consommés en monothérapie lors de l´automédication. Ceci concorde avec les résultats des travaux menés au Brésil [25] et au Burkina-Faso [32] dans lesquels les auteurs ont observé que la substance médicamenteuse la plus utilisée par les patients en automédication était le paracétamol. En revanche, une étude menée à Dakar au Sénégal révèle que la chloroquine (86%) était la plus utilisée en automédication [44]. L´usage dominant du paracétamol (antalgique et antipyrétique) et du Coartem (un antipaludéen) par nos enquêtés semblent logique par rapport aux maladies ou symptômes les poussant à cette pratique, à savoir le paludisme et les céphalées. Notons également qu´il a été remarqué que la forme pharmaceutique la plus prédominante est le comprimé (8%). D´autres auteurs avaient aussi établi la prépondérance de cette forme pharmaceutique dans l´usage des médicaments en automédication [6]. Pour l´inefficacité du médicament (54,9%), 85,5% des étudiants procèdent au changement du médicament. Ce changement pourrait s´expliquer également par le choix soit d´un médicament inapproprié, une posologie incorrecte mais aussi et surtout un mauvais diagnostic fait dès le départ. Un nombre suffisant des travaux avaient déjà fustigés comme abus lors de l'automédication, la non-maitrise des indications et des posologies [21,45]. Par ailleurs, 66% des sujets affirment avoir été obligés de consulter le médecin lors du second l´échec de l´automédication et 94,1% d´entre eux indiquent au médecin les médicaments consommés lors de l´automédication.

Une automédication associant plusieurs médicaments est observée chez les sujets enquêtés à hauteur de 88,5%. Cette habitude d´association des médicaments dans l´automédication est aussi observée dans une étude menée par [46]. La prise concomitante du Fansidar-Coartem (17,2%) a été la plus observée dans cette étude. Cependant, du point de vue pharmacologique, cette association n´est pas justifiée étant donné que les deux médicaments sont des antipaludéens [47]. Dans la présente étude, 37,4% des sujets interrogés recourent à l´automédication par des plantes médicinales ; cette prévalence trouvée est inférieure à celle des études effectuées à Lubumbashi en 2015 [6] et en 2020 [46]. Ceci peut s´expliquer par le fait probable que la médecine traditionnelle est prise avec plus des considérations à Lubumbashi que dans la ville d´Uvira. Selon la littérature consultée, des données en rapport avec l´exploitation en automédication de la médecine traditionnelle dans la ville d´Uvira sont encore moins nombreuses. Ce travail ouvre ainsi une brèche de cette optique. Néanmoins le recours à cette médecine reste une réalité en République Démocratique du Congo en générale [48] et dans la province du Sud-Kivu en particulier, y compris la ville d´Uvira [13]. D´ailleurs, il s´avère que 80% de la population mondiale fait recourt à la médecine traditionnelle pour essayer de répondre à ses besoins de santé [13]. Et aussi, une étude menée au Nord-Kivu, dans la ville de Goma, en République Démocratique du Congo, avait rapporté une prévalence de 57% de la population recourant à la médecine traditionnelle [10]. Notons que l´absence de la corrélation ou dépendance statistique entre les paramètres socio-démographiques et la prévalence de l´automédication observée dans cette étude serait expliquer par le fait que la quasi-totalité de personnes ayant participé à cette enquête recours à cette pratique. Le non-inclusion de toute la population d´Uvira dans cette étude constituerai la principale limite. De ce fait, le présent rapport scientifique ne donne pas l´image complète de la pratique de l´automédication dans toute la population d´Uvira. Néanmoins, l´étude pose un premier jalon dans ce domaine de la recherche dans la zone de l´étude.

## Conclusion

Cette étude confirme la pratique extrême de l´automédication par la quasi-totalité des étudiants enquêtés d´Uvira en RD Congo souvent dans la lutte contre les maladies courantes de la région, en particulier le paludisme. L´acquisition de médicaments sans ordonnance médicale serait l´un de facteurs prédisposants. Ainsi, il est indispensable que des efforts quant à l'usage rationnel des médicaments soient conjugués entre les personnels de santé et les décideurs politiques afin d'éradiquer toute sorte de pratique d´automédication. L´accès à certain médicament devrait être conditionné par une ordonnance médicale. Aussi, étant donné que le recours aux plantes médicinales lors de l´automédication est observé à une échelle non négligeable dans cette étude, la mise au point d'une interface de collaboration entre la médecine traditionnelle et la biomédecine serait louable dans la ville d´Uvira. En définitif, que les décideurs politiques en connivence avec ceux en matière de santé envisagent de développer et de mettre en œuvre des programmes sur les risques d'automédication, et le renforcement du contrôle et de la surveillance de la vente de médicaments. Rendre les soins de santé primaire accessible aux étudiants de la ville d´Uvira serait également une voie efficace pour diminuer sensiblement l'automédication dans le milieu estudiantin.

### Etat des connaissances sur le sujet

- *L´automédication est responsable de plusieurs méfaits de la santé sur le plan mondial;*
- *Le recours à l´automédication chez les étudiants est souvent relié à la prétention des connaissances des médicaments*.

### Contribution de notre étude à la connaissance

- *La prévalence de l´automédication chez les étudiants dans la ville d´Uvira est très élevée;*
- *Le manque des moyens financiers pour le soin de santé est à la base de cette pratique;*
- *Les pharmaciens constituent les principales sources d´informations pour la pratique de l´automédication*.

## Remerciements

Les auteurs adressent leurs remerciements à tous les enquêteurs ayant participé à la collecte de données de cette étude. Il s'agit des étudiantes en Sciences biomédicales de la Faculté de Médecine de l'Université Notre Dame Tanganyika (République Démocratique du Congo) dont les noms suivent: Munguakonkwa Chombo Daniella et Rashidi Fatyma Meddy.
